# Carprofen for perioperative analgesia causes early anastomotic leakage in the rat ileum

**DOI:** 10.1186/1746-6148-8-247

**Published:** 2012-12-27

**Authors:** Rozemarijn J van der Vijver, Cees JHM van Laarhoven, Roger MLM Lomme, Thijs Hendriks

**Affiliations:** 1 Department of Surgery, Radboud University Nijmegen Medical Centre, PO Box 9101, 6500 HB Nijmegen, The Netherlands

**Keywords:** NSAID, Carprofen, Rat, Anastomosis, Wound healing, Ileum

## Abstract

**Background:**

There is increasing evidence that perioperative use of NSAIDs may compromise the integrity of intestinal anastomoses. This study aims to characterize the negative effects of carprofen on early anastomotic healing in the rat ileum.

**Results:**

In 159 male Wistar rats an anastomosis was constructed in the ileum. In experiment 1 eighty-four rats were divided over control and experimental groups, which received daily buprenorphine or carprofen, respectively, as an analgesic and were killed on day 1, 2 or 3 after surgery. In experiment 2 three groups of 15 rats received carprofen either immediately after surgery or with a delay of 1 or 2 days. Animals were killed after 3 days of carprofen administration. In experiment 3 three groups of 10 rats received different doses (full, half or quarter) of carprofen from surgery.

In significant contrast to buprenorphine, which never did so, carprofen induced frequent signs of anastomotic leakage, which were already present at day 1. If first administration was delayed for 48 hours, the leakage rate was significantly reduced (from 80 to 20%; p = 0.0028). Throughout the study, the anastomotic bursting pressure was lowest in animals who displayed signs of anastomotic leakage. Loss of anastomotic integrity did not coincide with reduced levels of hydroxyproline or increased activity of matrix metalloproteinases.

**Conclusions:**

Carprofen interferes with wound healing in the rat ileum at a very early stage. Although the mechanisms responsible remain to be fully elucidated, one should be aware of the potential of NSAIDs to interfere with the early phase of wound repair.

## Background

The use of analgetics in veterinary medicine is still relatively low, but adequate management of animal pain is becoming increasingly important in companion, farm and laboratory animals [1,2].

Human pain control strategies using opiods or non-steroidal anti-inflammatory drugs (NSAIDs) can be applied to animals, because most animals possess neuronal mechanisms for pain perception which are similar to humans [3]. NSAIDs inhibit cyclo-oxygenases (COXs), enzymes which catalyze the first two steps in prostanoid biosynthesis. These enzymes occur as isoforms COX-1 and COX-2. COX-1 is expressed constitutively in housekeeping functions, whereas COX-2 is expressed mainly in response to external stimuli. One widely used NSAID in veterinary medicine is carprofen, which is believed to possess a certain preference for COX-2 [4] and is suitable for pain relief. It is mostly used in dogs and cats but also in cattle and in laboratory animals. If carprofen is used as an analgetic during and after surgery of the intestine in rats the healing of ileal, but not of colonic, anastomoses is seriously compromised [5]. A similar effect was found previously for celecoxib, another inhibitor with a certain degree of specificity for COX-2 [6]. Such a negative effect could have implications for the use of carprofen in veterinary practice and laboratory animals, particularly if used in the peri-operative period.

The present experiments were conducted to expand our earlier preliminary findings [5] and further investigate the effects of carprofen on the healing of anastomoses in the small intestine of the rat.

## Methods

### Study design

Hundred and fiftynine male Wistar rats weighing 250–290 g (Charles River, Sulzfeld, Germany) were housed 2 per cage and accustomed to laboratory conditions for five days before the start of the experiment. All rats underwent intestinal resection and an anastomosis was constructed in the ileum. Animals were observed closely and weighed daily and had free access to water and standard rodent chow (Hope Farms, Woerden, The Netherlands) throughout the entire experimental period. When observing the rats attention was paid to activity, colour and aspect of nose, presence of diarrhoea, aspect of fur and tenderness of the abdomen. If the rats scored as abnormal on all of these points they were taken out of the experiment, always after consultation of one of the experienced biotechnicians.

In experiment 1, 84 rats were randomly divided into two cohorts of 42 rats, each consisting of three groups of 14 animals to be killed on day 1, 2 or 3 after surgery. The controls were administered buprenorphine and the experimental animals received carprofen as an analgetic (Table 1). Ten rats from each group were analysed for wound strength and the remaining animals were used for histology.

**Table 1:** Characteristics of experiment 1

	**Buprenorphine**			**Carprofen**		
**Day of termination**	**1**	**2**	**3**	**1**	**2**	**3**
**Number operated at day 0**	**14**	**14**	**14**	**14**	**14**	**14**
**Premature death (day)**	**0**	**1 (0)**	**0**	**0**	**0**	**1 (2)**
**Anastomotic leakage**	**0**	**0**	**0**	**6(p = 0.02)**	**8(p = 0.002)**	**11(p = <0.0001)**

After termination 10 animals from each group were analysed for wound strength and the remaining animals for histology. At obduction, special attention was paid to signs of anastomotic leakage. P-values denote significant differences between each carprofen group and the corresponding buprenorphine group (Fisher’s two-sided test).

In experiment 2, 45 rats were randomly divided over three equal groups. All received carprofen for three days, starting immediately after surgery or 1 or 2 days afterwards. Animals in the three groups were killed on day 3, 4 or 5 after surgery, respectively (Table 2). Twelve rats from each group were analysed for wound strength and the remaining animals were used for histology.

**Table 2:** Characteristics of experiment 2

	**Administration of carprofen from day 0**	**Administration of carprofen from day 1**	**Administration of carprofen from day 2**
**Day of termination**	**3**	**4**	**5**
**Number operated at day 0**	**15**	**15**	**15**
**Premature death (day)**	**0**	**2 (3,3)**	**0**
**Anastomotic leakage**	**12**	**7**	**3(p = 0.0028)**

After termination 12 animals in each group were analysed for wound strength and the remaining animals for histology. Signs of anastomotic leakage were noted at obduction. P-value denotes a significant difference with the group administered carprofen from day 0 (Fisher’s two-sided test).

In experiment 3, 30 rats were divided into three equal groups which each received a different daily (full, half or quarter, see below) dose of carprofen from the day of surgery. All animals were killed at day 3 (Table 3) and analysed for wound strength.

**Table 3:** Characteristics of experiment 3

	**Full dose carprofen**	**Half dose carprofen**	**Quarter dose carprofen**
**Number operated at day 0**	**10**	**10**	**10**
**Day of termination**	**3**	**3**	**3**
**Premature death (day)**	**1 (2)**	**0**	**1 (3)**
**Anastomotic leakage**	**9**	**8**	**6**

All animals, except those who died prematurely, were terminated at day 3 and analysed for wound strength. Signs of anastomotic leakage at obduction were noted.

The Animal Ethics Review Committee of the Radboud University Nijmegen approved the study (RU-DEC 2011–015 and 2011–127).

### Surgery and analgesics

Procedures were performed under semi sterile conditions using a Zeiss operation microscope (Carl Zeiss AG, Oberkochen, Germany). Animals were anesthetized by use of a mixture of isoflurane, oxygen and nitrogen, while breathing spontaneously through a mask.

A midline laparotomy was performed and in each rat a 0.5-cm segment was resected from the distal ileum, 15 cm proximal to the coecum. Ileal continuity was restored by constructing an end-to-end anastomosis with 8 single-layer, inverting, interrupted sutures (Ethilon 8–0; Ethicon, Norderstedt, Germany).

The abdominal wall was closed with a running suture (Vicryl 3–0; Ethicon, Norderstedt, Germany). The skin was closed with staples. During operations, body temperature was kept at 38°C using a heating pad and a lamp. Intestines were covered with gauze pads soaked with 0.9% NaCl to minimize desiccation. To prevent dehydration, 10 ml of 0.9% NaCl was administered subcutaneously after the operation.

All rats in experiment 2 and 3 and the rats in the control group of experiment 1 were administered buprenorphine (Temgesic, Schering Plough, Houten, the Netherlands), 0.02 mg/kg subcutaneously every 12 h for 48 h the first dose was administered just prior to surgery. This constitutes the analgesic regimen routinely given in our intestinal anastomosis model. In experiment 1, rats in the experimental group received only carprofen (Rimadyl, Pfizer Animal Health, Capelle aan de IJssel, the Netherlands), 5 mg/kg subcutaneously daily immediately before surgery and on days 1 and 2. In experiment 2 animals received 3 daily doses of carprofen (5 mg/kg) starting on the day of surgery or 1 or 2 days afterwards. The rats in experiment 3 received carprofen in different doses (5, 2.5 and 1.25 mg/kg, respectively) on the day of surgery and the next 2 days.

### Necropsy and analysis of wound strength

The rats were killed by CO/CO_2_ asphyxiation. The abdomen was inspected and particular attention was paid to signs of anastomotic leakage defined such as macroscopic dehiscence of the anastomosis, the presence of faecal peritonitis or a puncture in the anastomotic line with or without an abscess near it. If necessary (in order to resect the segment) adhesions were dissected carefully without manipulation of the anastomosis proper. Segments containing the anastomoses were resected en bloc.

To measure bursting pressure, the segments were infused (2 ml/min) with 0.9% NaCl containing methylene blue. The maximum pressure (mmHg) recorded immediately before sudden loss of pressure was taken as the bursting pressure. The site of rupture (within or outside the anastomotic line) was noted. Subsequently, the same segments were placed in a tensiometer, and the breaking strength (g) was measured [7]. The anastomotic segments were carefully cleaned from any adhering tissue and 5 mm samples, containing the suture line in the middle, were frozen in liquid nitrogen and stored at −80°C until further processing.

### Biochemical analysis

After weighing, tissue samples were frozen, lyophilized and pulverized. The hydroxyproline content, as a measure of the collagen content, was measured in experiment 1 by high-performance liquid chromatography (HPLC) after hydrolysis with 6 N hydrochloric acid and coupling to dabsyl-chloride.

In experiment 1gelatin zymography was performed. Tissue extracts were prepared using a buffer containing 1% (v/v) Triton X-100. The protein concentration of the extracts was measured using the bicinchoninic acid reagent. All tissue samples were stored at −80°C until zymography. The techniques for preparation and electrophoresis of the gels and quantification of the various enzyme activities have been described previously [8].

### Histology

Intestinal samples of approximately 1 cm containing the entire anastomosis in the middle were carefully collected en bloc, opened at the mesenteric side, washed gently in 0.9% NaCl and spread out in a cassette for paraffin-embedding. From paraffin-embedded tissues, 4 μm sections were prepared and stained with hematoxylin and eosin (H&E). The presence of COX-1 and COX-2 protein in healing anastomoses was visualized by immunohistochemistry. From the paraffin-embedded tissues 4 μm sections were prepared and stained as described previously [6]. COX-1 and COX-2 proteins were visualized using a rabbit polyclonal antiserum against human COX-1 and COX-2 (Cayman Chemical, An Arbor, MI, USA).

### Statistics

Comparison between more than two groups was performed using a One-way Analysis of Variance (ANOVA). Comparisons between two groups or, within one group, between values at two different days were performed with a two-tailed unpaired *t*-test. Results were considered statistically significant at p < 0.05.

## Results

### Presence of COX during normal healing

**Figure 1:**
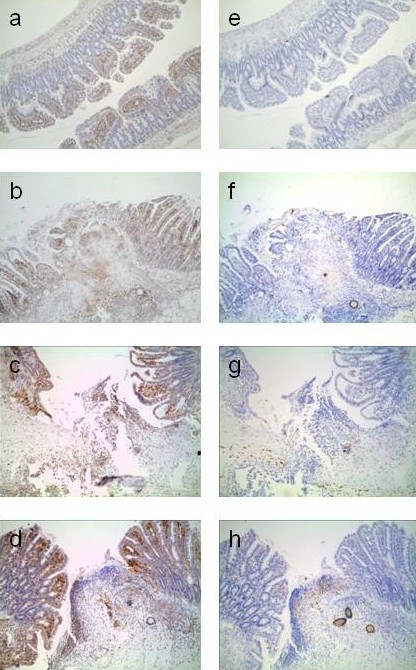
**Presence of COX-1 and COX-2 during normal healing. **Anastomotic segments from the buprenorphine group of experiment 1 were immunostained for COX-1 (panels on the left) and COX-2 (panels on the right). Panels represent uninjured intestine (**a** and **e**) and tissue segment with the anastomosis in the middle and the mucosal layer on top (at a magnification of approximately x100) at day 1 (**b** and **f**), 2 (**c** and **g**) and 3 (**d** and **h**) after surgery.

Both COX-1 and COX-2 are expressed during normal healing, if buprenorphine is used for peroperative pain relief. Figure 1 shows COX-1 to be present in undamaged intestine and also at day 1, 2 and 3 after surgery (Figure 1 panels a-d). COX-2 is absent in undamaged intestine but appears at day 1 and, more strongly, at days 2 and 3 (Figure 1panels e to h).

### Experiment 1: carprofen versus buprenorphine

All animals lost weight after operation. In the buprenorphine group the mean (+ SEM) relative weight (vs weight at operation) was 91 ± 1% at day 3. The corresponding value in the carprofen group was 94 ± 2% (p = 0.0356). One rat from the buprenorphine group died immediately after surgery of no discernible reason. In the carprofen group another rat died prematurely from anastomotic leakage. No signs of anastomotic leakage were observed in the buprenorphine groups. These signs were found increasingly with time, and significantly (p < 0.05) more frequently at all days, in the carprofen groups (Table 1).

**Figure 2:**
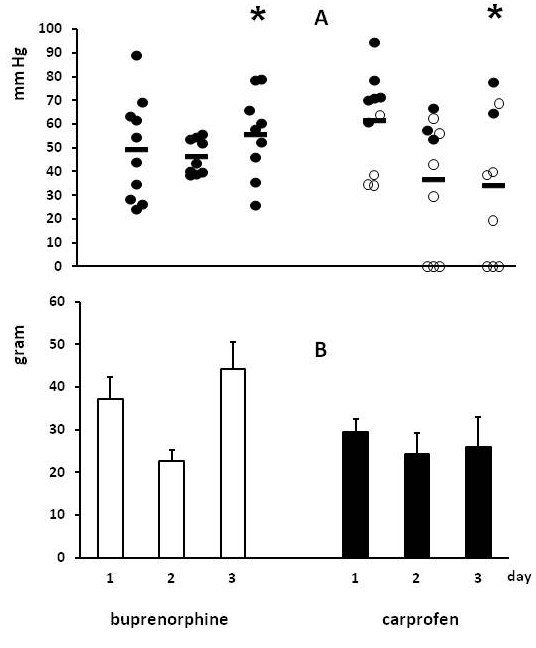
**Anastomotic strength in experiment 1. **Animals received either buprenorphine or carprofen from the day of operation and were terminated at day 1, 2 or 3 after operation. Panel **A **gives the individual bursting pressures (and means as horizontal bars) for all animals: *black circle *denotes an absence of signs of anastomotic leakage and ○ the presence of such signs. The bursting site was always in the anastomotic line. In panel **B **bars represent mean (+ SEM) for breaking strength.

The anastomotic bursting pressure is depicted in Figure 2A. The biggest difference between groups was observed at day 3 where values in the buprenorphine group averaged 56 ± 6 (SEM) mm Hg and in the carprofen group 34 ± 10 mm Hg (p = 0.0915). Clearly, in the carprofen groups the bursting pressure was lowest in animals who displayed signs of anastomotic leakage. At day 3, the average anastomotic breaking strength (Figure 2B) was also lower (p = 0.0729) in the carprofen group than in the buprenorphine group, at 26 ± 7 vs 44 ± 6 g, respectively.

**Figure 3:**
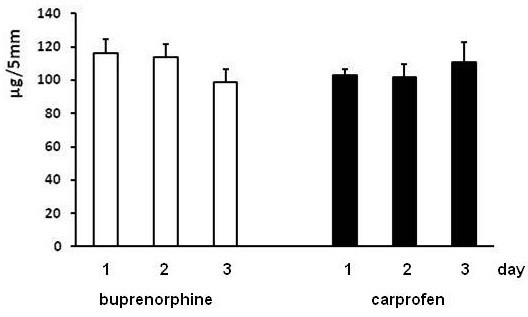
**Anastomotic hydroxyproline content in experiment 1. **Bars represent mean and SEM (in μg hydroxyproline/5 mm tissue) in anastomotic segments from both the buprenorphine and the carprofen groups at the various days after operation.

Gelatin zymography revealed the presence of both pro- and active MMP-2 and −9 in anastomotic extracts. At days 1 and 2 no differences were found for any of these activities between buprenorphine and carprofen groups (data not shown). At day 3, total activities (in arbitrary units/sample) for both pro-MMP-2 and active MMP-2 were higher in the buprenorphine groups: 571 ± 62 vs 313 ± 35 (p = 0.0038) and 223 ± 21 vs 133 ± 9 (p = 0.0024) respectively. The anastomotic hydroxyproline content did not significantly differ between groups at any of the days (Figure 3).

### Experiment 2: delayed administration of carprofen

**Figure 4:**
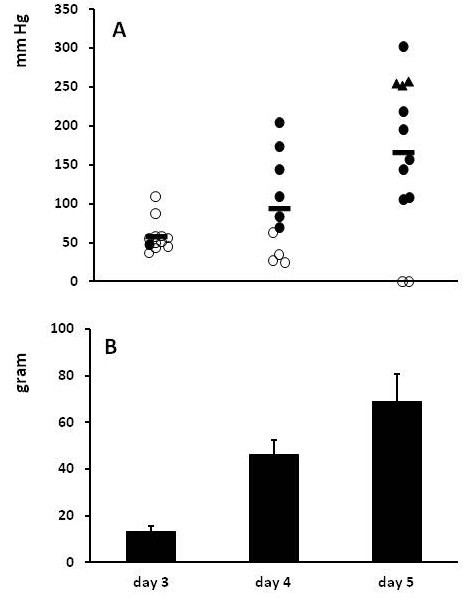
**Anastomotic strength in experiment 2. **Animals received carprofen beginning at the day of operation or the first or second day thereafter and were terminated 3 days later. Panel **A **gives the individual bursting pressures (and means as horizontal bars) for all animals: *black circle *denotes an absence of signs of anastomotic leakage and ○ the presence of such signs, while *black triangle* indicates that the bursting site was outside the anastomotic line. In panel **B** bars represent mean (+ SEM) for breaking strength.

Weight loss was similar to that observed in experiment 1, with the relative weight at day 3 averaging 93, 91 and 93% in the groups receiving carprofen from day 0, 1 or 2, respectively. Two rats which received carprofen from day 1 after surgery died prematurely on day 3 due to anastomic leakage. As before, signs of anastomotic leakage were seen frequently if carprofen was given from day 0 (Table 2). Delaying the first gift reduced leakage, significantly so if carprofen was only given from day 2 onwards, although in that group signs were still observed in 20% of the animals after termination at day 5. Anastomotic strength did increased with time. Despite the fact that the animals also received a three day course of carprofen both anastomotic bursting pressure and breaking strength at day 5 were considerably and significantly (p < 0.001) higher than at day 3 (Figure 4). Again, the bursting pressure was lowest in animals who displayed signs of anastomotic leakage.

### Experiment 3: different dosage of carprofen

The weight loss was similar to that in the preceding experiments, with a mean relative weight (vs weight at operation) at day 3 of 94, 96 and 94% in the groups receiving full, half or quarter doses of carprofen, respectively. One rat died prematurely of anastomotic dehiscence in the group which received the full dose of carprofen and another one, of unknown reasons, in the group that received a quarter dose.

Signs of anastomotic leakage were abundant in all groups (Table 3). Although the incidence decreased with dose, this effect remained non-significant with the current cohort size.

**Figure 5:**
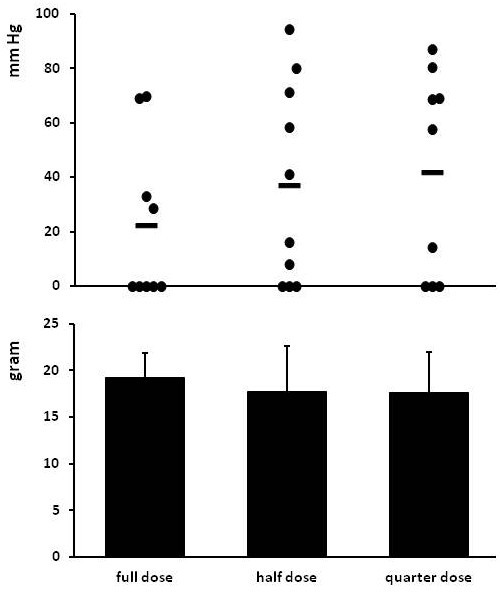
**Anastomotic strength in experiment 3. **Animals received different doses of carprofen from the day of operation and were terminated at day 3 after operation. Panel **A **gives the individual bursting pressures (and means as horizontal bars) for all animals: *black circle *denotes an absence of signs of anastomotic leakage and ○ the presence of such signs. The bursting site was always in the anastomotic line. In panel **B **bars represent mean (+ SEM) for breaking strength.

The average bursting pressure rose slightly with decreasing dosage of carprofen but did not differ significantly between groups (Figure 5). Animals without signs of anastomotic leakage showed the highest anastomotic bursting pressure. Breaking strength was similar in all groups.

## Discussion and conclusion

Carprofen interferes with early repair of ileal anastomoses in the rat. Signs of anastomotic leakage comprise the main outcome parameter in this study. If carprofen is used from the day of operation, these signs are already abundantly present 24 hours later. Delaying the first gift of carprofen for 48 hours significantly reduces signs of leakage, without completely preventing them. Reducing the dose fourfold lowers the incidence of this complication, but not significantly so.

NSAIDs are commonly used in small animals and equine practice for peri-operative pain relief because of their analgetic and anti-inflammatory features. NSAIDs inhibit the family of COX enzymes. COX-2 is expressed mainly in response to external stimuli and is postulated to be involved in essentially pathological conditions such as inflammation, pain and fever [9]. Selective inhibitors of COX-2 supposedly allow specific targeting of inflammatory disease processes, without disruption of normal homeostatic mechanisms that accounts for many side-effects of non-selective NSAID therapy. Both COX-1 and −2 are believed to be involved in wound healing [10,11] and Figure 1 demonstrates their presence in the healing anastomosis. There is a growing understanding that NSAIDs, and especially those with a certain preference for COX-2, may increase the risk of anastomotic leakage [12,13]. Carprofen is such a drug that is used in domestic animals [14], but is also effective as an analgesic in laboratory animals undergoing laparotomy [15]. Recent evidence shows that its long-term use can inhibit bone healing in dogs [16], but nothing is known about its potential effects on soft tissue healing. Recent incidental reports suggest that carprofen administration to dogs alters functions of platelets, which are relevant to the repair sequence [17]. Also, carprofen has been shown to compromise the integrity and barrier function of the gastrointestinal mucosa in dogs [18]. The present data, which expand on a previous short communication [5] unequivocally demonstrate that carprofen can interfere significantly with anastomotic integrity in the rat small bowel.

While signs of leakage in the carprofen group greatly surpass those in the buprenorphine group (Table 1), the average anastomotic strength is not dramatically reduced (Figure 1). It seems likely that in the majority of animals the leakage can be contained, e.g. by the formation of fibrinous adhesions. This way, some degree of strength is restored to the anastomotic segment. On the whole though, throughout the three experiments anastomoses which display signs of leakage at necropsy display lower bursting pressures than those that are free of such signs. These findings raise the question what the fate would have been of the animals which survive until day 3 or later, but which show signs of leakage. Most likely, those with zero bursting pressure, thus in fact with a gap in the suture line at that time, will eventually die from secondary peritonitis. However, the possibility cannot be excluded that in some cases a fibrinous adhesion barrier will prevent full feacal leakage into the abdominal cavity. The resulting subclinical leakage could eventually allow complete healing of the intestinal wall. just as the majority of animals with anastomotic strength compatible with normal repair at day 3 may be expected to survive and show increasing strength in the proliferative phase of healing (as observed in the animals in experiment 2).

From the data presented it is clear that, whatever mechanism is responsible for leakage to occur, the origin of the phenomenon must lie within the first days after operation. It is not confined to the first 24, or even 48, hours since in the group where carprofen was first given at day 2, 20% of the animals still showed proof of leakage 3 days later (Table 2). Thus, carprofen somehow affects the inflammatory phase of healing, where anastomotic integrity is determined by the capacity of the existing submucosal matrix to retain sutures. Induction of massive matrix degradation seems unlikely since hydroxyproline (collagen) levels in the anastomotic segments remain unaffected. Also, total activities of MMP-2 and MMP-9 remain unchanged during the first 2 days. These results do not exclude the possibility of limited and local matrix degradation, e.g. around the sutures, by one of the other enzymes from the MMP family. Still, it is believed that COX-2 inhibitors generally suppress MMP activity [19].

It has been suggested that COX-2 plays a regulatory role in maintaining gastrointestinal barrier function and motility and is needed to maintain small bowel integrity [20,21].

Interestingly, the effect described here does not occur in colonic anastomoses [5] while the highest level of COX-2 expression in normal uninjured rats is located on the ileal side of the ileocaecal junction [22]. The question then arises if the carprofen effect is mediated specifically through COX-2 and if it is species specific. It has been suggested that determining the exact specificity of any inhibitor for either of the COX enzymes is frought with practical difficulties and may depend on the test and the laboratory that uses it [4]. Still, carprofen seems to possess a certain degree of specificity for COX-2, although it may vary between species. Thus, as yet the question remains if these findings are limited to laboratory animals or that they can also occur in dogs and cats. Using a commercially available formulation and the manufacturer’s recommended dose it seems likely that carprofen also produces significant inhibition of COX-1 [4]. Very recent data from our own laboratory suggest that diclofenac, another inhibitor with specificity for COX-2, shows effects similar to carprofen, while naproxen, with a suspected lesser specificity for COX-2, leaves ileal anastomoses intact (unpublished results), Older data indicate increased complications in rats with anastomoses in both ileum and colon after preoperative administration of either ibuprofen or indomethacin [23]. It thus remains to be determined if preference for COX-2 is essential for a drug to exert the negative effects observed.

It is necessary to gain knowledge about the potential drawbacks of NSAID’s like carprofen, as they are rapidly becoming cornerstones in peri-operative pain relief and are able to minimize post-operative opioid requirement in veterinary practice and in experimental studies [2-24]. The findings reported here may also be relevant to the human situation where NSAIDs are frequently used after gastro-intestinal surgery and are even incorporated in protocols for fast track surgery, despite emerging evidence that they may affect repair [12,13].

Thus, we conclude that carprofen interferes with wound healing in the rat ileum at a very early stage. If it does not kill the animal, at least it renders it more vulnerable to second hits after initial surgery. Although the mechanisms responsible remain to be fully understood, there appears to be an increasing body of evidence which suggests that one should be aware that NSAIDs may affect the outcome of the wound healing sequence.

## Competing interests

None of the authors of this paper has a financial or personal relationship with other people or organisations that could inappropriately influence or bias the content of the paper.

## Authors’ contributions

RvdV designed the experiment, collected data, analysed data and drafted the manuscript. CvL designed the experiment and drafted the manuscript. RL collected and analysed data. TH designed the experiment, analysed data and drafted the manuscript. All authors read and approved the final manuscript.
